# Molecular and morphological evidence reveal a new genus and species in Auriculariales from tropical China

**DOI:** 10.3897/mycokeys.35.25271

**Published:** 2018-06-20

**Authors:** Hai-Sheng Yuan, Xu Lu, Cony Decock

**Affiliations:** 1 CAS Key Laboratory of Forest Ecology and Management, Institute of Applied Ecology, Chinese Academy of Sciences, Shenyang 110164, China; 2 University of the Chinese Academy of Sciences, Beijing 100049, China; 3 Mycothèque de l’Université catholique de Louvain (MUCL, BCCMTM), Earth and Life Institute – Microbiology (ELIM), Université catholique de Louvain, Croix du Sud 2 bte L7.05.06, B-1348 Louvain-la-Neuve, Belgium

**Keywords:** *Grammatus labyrinthinus*, ITS and nLSU, lignicolous fungi, phylogeny, taxonomy

## Abstract

*Grammatus labyrinthinus* **gen. et sp. nov.** is proposed based on DNA sequences data and morphological characteristics. It is known so far from southern, tropical China. The new species is characterised by an annual, resupinate basidiocarp with a shallow, subporoid hymenophore, a hymenium restricted to the bottom of the tubes, a dimitic hyphal system, presence of encrusted skeletocystidia and dendrohyphidia, longitudinally septate basidia and smooth, oblong-ellipsoid to cylindrical, acyanophilous basidiospores. Phylogenetic analyses based on ITS + nLSU DNA sequences data indicate that *G. labyrinthinus* belongs to Auriculariaceae in which it has an isolated position. Phylogenetic inferences show *G. labyrinthinus* to be related to *Heteroradulum*. However, the ITS sequences similarity between *G. labyrinthinus* and *H. kmetii*, the type species of *Heteroradulum*, were 89.84% and support the establishment of the new genus. Inversely, *Heteroradulum semis* clustered with *G. labyrinthinus* with strong support and it is transferred to *Grammatus*.

## Introduction

Auriculariales was established by Schroeter (1889) and originally accommodated species which, with transversely septate basidia, is also known as auricularioid basidia. Based on the micromorphology and ultra-structure of the septal pore and the spindle pole body, Bandoni (1984) redefined Auriculariales, in order to accommodate all heterobasidiomycetes with continuous parenthesomes, transversely or longitudinally septate basidia and hyphal haploid stages. Currently, one family, Auriculariaceae is accepted and 198 species are distributed into 32 genera of Auriculariales (Kirk et al. 2008).

Auriculariaceae are diverse as long as their basidiocarp consistency (flesh gelatinous, wax-like and corky) and hymenophore structures (smooth, plicate, hydnoid and poroid) are concerned. According to the Dictionary of the fungi (10^th^ edition), the family includes 7 genera: *Auricularia* Bull., *Eichleriella* Bres., *Elmerina* Bres., *Exidia* Fr., *Exidiopsis* (Bref.) Möller, *Fibulosebacea* K. Wells & Raitv., *Heterochaete* Pat. and 112 species (Kirk et al. 2008). Two other genera related to *Elmerina*, *viz. Aporpium* Bondartsev & Singer and *Protodaedalea* Imazeki, were also described. However, the phylogenetic position of the type species of *Elmerina* is still unclear (Sotome et al. 2014). The latest study of Auriculariales using ITS + nLSU DNA sequences data introduced or revalidated several new genera, *viz. Amphistereum* Spirin & V. Malysheva, *Sclerotrema* Spirin & V. Malysheva, *Heteroradulum* Lloyd and allowed the inclusion of *Exidia glandulosa* (Bull.) Fr., *Hirneolina hirneoloides* (Pat.) Pat., *Tremellochaete japonica* (Lloyd) Raitviir in Auriculariaceae (Malysheva and Spirin 2017).

Molecular phylogeny had been widely used to investigate phylogenetic relationships amongst the genera and species in Auriculariales (Swann and Taylor 1993, Berres et al. 1995, Weiß and Oberwinkler 2001, Kirschner and Chen 2004, Wells et al. 2004, Kirschner et al. 2010, 2012, Zhou and Dai 2013, Sotome et al. 2014, Malysheva and Spirin 2017). These phylogenetic contributions provided a general overview of Auriculariales and show strong support at the species level, but so far, not all the deeper nodes received high support from molecular evidence.

China is very rich in wood-decaying fungi and extensive studies on species diversity, taxonomy, ecology and phylogeny of wood-decaying fungi have been carried out recently (Yuan and Dai 2008; Dai 2010, 2011, 2012; Yuan et al. 2015). During a continuous survey of wood-decaying Basidiomycetes in the Yunnan Province, tropical China, a species of Auriculariaceae was collected but could not be confidently identified to any known species. The morphology characteristics suggested they represent an undescribed genus in Auriculariaceae. The aim of this paper is to clarify the taxonomic status of the genus and to describe new taxa.

## Materials and methods

*Morphological studies*. Specimens are deposited at the herbarium of Institute of Applied Ecology, Chinese Academy of Sciences (IFP). Microscopic procedures follow Yuan and Qin (2018). The microscopic studies were made from sections mounted in Cotton Blue (CB): 0.1 mg aniline blue dissolved in 60 g pure lactic acid; CB+/– = cyanophilous / acyanophilous. Amyloid and dextrinoid reactions were tested in Melzer’s reagent (IKI): 1.5 g KI (potassium iodide), 0.5 g I (crystalline iodine), 22 g chloral hydrate, aq. dest. 20 ml; IKI– = neither amyloid nor dextrinoid reaction. KOH (5%) was used as a mounting reagent. Sections were studied at magnifications up to 1000× using a Nikon Eclipse E600 microscope and phase contrast illumination and dimensions were estimated subjectively with an accuracy of 0.1 µm. For spore measurements, the apiculus was excluded. In presenting the variation of spore size, 5% of the measurements at each end of the range are given in parentheses. The following abbreviations are used in the text: L = mean spore length, W = mean spore width, Q = range of length/width ratios for studied specimens and n = total number of spores measured from a given number of specimens. Special colour terms are from Petersen (1996).

*Molecular procedures and phylogenetic analyses.* The fungal taxa and strains used in this study are listed in Table 1. Phire Plant Direct PCR Kit (Finnzymes, Finland) procedure was used to extract total genomic DNA from the fruiting body and for the polymerase chain reaction (PCR). PCR amplification was confirmed on 1% agarose electrophoresis gels stained with ethidium bromide (Stöger et al. 2006). DNA sequencing was performed at Beijing Genomics Institute (BGI). All newly generated sequences have been submitted to GenBank and are listed in Table 1.

**Table 1.** DNA sequences used in the present study.

Species	Collector/herbarium number	ITS GenBank#	LSU GenBank#	Source
*Amphistereum leveilleanum*	Lentz FP-106715 (CFMR)	KX262119	KX262168	Malysheva and Spirin 2017
*A. schrenkii*	Burdsall 8476 (CFMR)	KX262130	KX262178	Malysheva and Spirin 2017
*Aporpium hexagonoides*	ML297 (TFM)	AB871754	AB871735	Sotome et al. 2014
*Auricularia cornea*	AU110	KF297960	KF297995	unpublished
*A. fuscosuccinea*	MW530	AB615231	AF291291	Weiß and Oberwinkler 2001
*A. mesenterica*	FO 25132	AF291271	AF291292	Weiß and Oberwinkler 2001
*A. mesenterica*	TUFC12805	AB915192	AB915191	Sotome et al. 2014
*A. polytricha*	TUFC12920	AB871752	AB871733	Sotome et al. 2014
*Basidiodendron caesiocinereum*	MW 320	–	AF291293	Weiß and Oberwinkler 2001
*B. eyrei*	MW 529	–	AF291296	Weiß and Oberwinkler 2001
*B. eyrei*	TUFC14484	AB871753	AB871734	Sotome et al. 2014
*Bourdotia galzinii*	FO 2278	–	AF291301	Weiß and Oberwinkler 2001
*Ductifera pululahuana*	KW 1733	–	AF291315	Weiß and Oberwinkler 2001
*Eichleriella alliciens*	Burdsall 7194 (CFMR)	KX262120	KX262169	Malysheva and Spirin 2017
*E. bactriana*	I. Parmasto (TAAM 96698)	KX262123	KX262172	Malysheva and Spirin 2017
*E. bactriana*	E. Parmasto (TAAM 104431)	KX262138	KX262186	Malysheva and Spirin 2017
*E. crocata*	E. Parmasto (TAAM 101077)	KX262100	KX262147	Malysheva and Spirin 2017
*E. crocata*	E. Parmasto (TAAM 125909)	KX262118	KX262167	Malysheva and Spirin 2017
*E. desertorum*	Ryvarden 49350 (O)	KX262142	KX262190	Malysheva and Spirin 2017
*E. flavida*	Ryvarden 49412 (H)	KX262137	KX262185	Malysheva and Spirin 2017
*E. leucophaea*	Barsukova (LE 303261)	KX262111	KX262161	Malysheva and Spirin 2017
*E. leucophaea*	Larsson 15299 (O)	KX262136	KX262184	Malysheva and Spirin 2017
*E. shearii*	USJ 54609	AF291284	AF291335	Weiß and Oberwinkler 2001
*E. sicca*	Miettinen 17349 (H)	KX262143	KX262191	Malysheva and Spirin 2017
*E. tenuicula*	Ryvarden 17599 (O)	KX262141	KX262189	Malysheva and Spirin 2017
*Elmerina caryae*	WD2207	AB871751	AB871730	Sotome et al. 2014
*E. caryae*	Dai 4549	JQ764652	JQ764631	Zhou and Dai 2013
*E. cladophora*	Wei 5621	JQ764659	JQ764634	Zhou and Dai 2013
*E. dimidiata*	O18238	JQ764663	JQ764640	Zhou and Dai 2013
*E. dimidiata*	O18261	JQ764664	JQ764641	Zhou and Dai 2013
*E. efibulata*	Dai 9322	JQ764669	JQ764647	Zhou and Dai 2013
*E. foliacea*	Yuan 5691	JQ764666	JQ764644	Zhou and Dai 2013
*E. hispida*	WD548 (TFM)	AB871768	AB871749	Sotome et al. 2014
*E. hispida*	E701	AB871767	AB871748	Sotome et al. 2014
*E. hispida*	Wei 5584	JQ764667	JQ764645	Zhou and Dai 2013
*Exidia glandulosa*	TUFC 34008	AB871761	AB871742	Sotome et al. 2014
*E. glandulosa*	MW 355	AF291273	AF291319	Weiß and Oberwinkler 2001
*E. pithya*	MW 313	AF291275	AF291321	Weiß and Oberwinkler 2001
*E. uvapsassa*	AFTOL-ID 461	DQ241776	AY645056	unpublished
*Exidiopsis calce*a	MW 331	AF291280	AF291326	Weiß and Oberwinkler 2001
*E. effusa*	Miettinen 19136 (H)	KX262145	KX262193	Malysheva and Spirin 2017
*E. grisea*	RK 162	AF291281	AF291328	Weiß and Oberwinkler 2001
*E. gris*ea	TUFC100049	AB871765	AB871746	Sotome et al. 2014
*E.* sp.	TUFC34333	AB871764	AB871745	Sotome et al. 2014
*E.* sp.	FO 46291	AF291282	AF291329	Weiß and Oberwinkler 2001
*Grammatus labyrinthinus*	Yuan 1759	KM379137	KM379138	This study
*G. labyrinthinus*	Yuan 1600	KM379139	KM379140	This study
*Heterochaete andina*	Lagerheim (FH, lectotype)	–	KX262187	Malysheva and Spirin 2017
*H. delicata*	TUFC33717	AB871766	AB871747	Sotome et al. 2014
*Heterochaetella brachyspora*	RK 96	–	AF291337	Weiß and Oberwinkler 2001
*Heteroradulum adnatum*	Ryvarden 23453 (O)	KX262116	KX262165	Malysheva and Spirin 2017
*H. deglubens*	LE 38182	KX262112	KX262162	Malysheva and Spirin 2017
*H. deglubens*	TAAM 064782	KX262101	KX262148	Malysheva and Spirin 2017
*H. kmetii*	Kmet (H, lectotype)	KX262124	KX262173	Malysheva and Spirin 2017
*H. kmetii*	Spirin 6466 (H)	KX262104	KX262152	Malysheva and Spirin 2017
*H. semis*	Miettinen 10618.1 (H)	KX262146	KX262194	Malysheva and Spirin 2017
*Myxarium grilletii*	RK 218	–	AF291349	Weiß and Oberwinkler 2001
*M. nucleatum*	ZP TRE2M	–	AF291351	Weiß and Oberwinkler 2001
*Protodontia subgelatinosa*	USJ 54661	–	AF291357	Weiß and Oberwinkler 2001
*Protomerulius africanus*	Ryvarden 9800 (O)	–	AF291358	Weiß and Oberwinkler 2001
*Pseudohydnum gelatinosum*	MW 298	–	AF291360	Weiß and Oberwinkler 2001
*Sclerotrema griseobrunneum*	Niemelä 2722 (H)	KX262144	KX262192	Malysheva and Spirin 2017
*Sistotrema brinkmannii*	Isolate 236	JX535169	JX535170	GenBank
*Tremellochaete japonica*	LE 303446	KX262110	KX262160	Malysheva and Spirin 2017
*Tremellodendropsis* sp.	USJ 54427	–	AF291375	Weiß and Oberwinkler 2001
*Tremiscus helvelloides*	MW 337	–	AF291377	Weiß and Oberwinkler 2001

Nuclear ribosomal RNA genes were used to determine the phylogenetic position of the new species. The internal transcribed spacer (ITS) regions were amplified with the primers ITS4 and ITS5 and the partial nLSU regions were amplified with primers LR7 and LR0R (White et al. 1990). The most similar sequences were searched for in GenBank NCBI (http://www.ncbi.nlm.gov) using the BLAST option and downloaded (Table 1). Sequences were aligned using ClustalX (Thompson et al. 1997) and the alignment was deposited in TreeBASE (http://treebase.org/treebase-web/) (submission ID: 22496). Identity/similarity between two sequences was calculated using the BioEdit v. 7.2.6 (Hall 2005). Maximum parsimony (MP), Maximum likelihood (ML) and Bayesian inference were applied to the ITS + LSU dataset. All characters were weighted and gaps were treated as missing data. Maximum parsimony analysis (PAUP* version 4.0b10) was used (Swofford 2002). Trees were inferred using the heuristic search option with TBR branch swapping and 1000 random sequence additions. Max-trees were set to 5000 and no-increase, branches of zero length were collapsed and all parsimonious trees were saved. Clade stability was assessed using a bootstrap (BT) analysis with 1,000 replicates (Felsenstein 1985). Maximum likelihood (ML) analysis was performed in RAxML v8.2.4 with GTR + I + G model (Stamatakis 2014). The best tree was obtained by executing 100 rapid bootstrap inferences and, thereafter, a thorough search for the most likely tree using one distinct model/data partition with joint branch length optimisation (Stamatakis et al. 2008). Bayesian analysis with the latest version of MrBayes 3.2.6 (Ronquist and Huelsenbeck 2003) implementing the Markov Chain Monte Carlo (MCMC) technique. The best-fit models (GTR + I + G) were selected by hLRT in MrModeltest 2.3 (Nylander 2004). Four simultaneous Markov chains were run starting from random trees and keeping one tree every 100^th^ generation until the average standard deviation of split frequencies was below 0.01. The value of burn-in was set to discard 25% of trees when calculating the posterior probabilities. Bayesian posterior probabilities were obtained from the 50% majority rule consensus of the trees kept.

## Results

### Phylogenetic analyses

**Figure 1.**
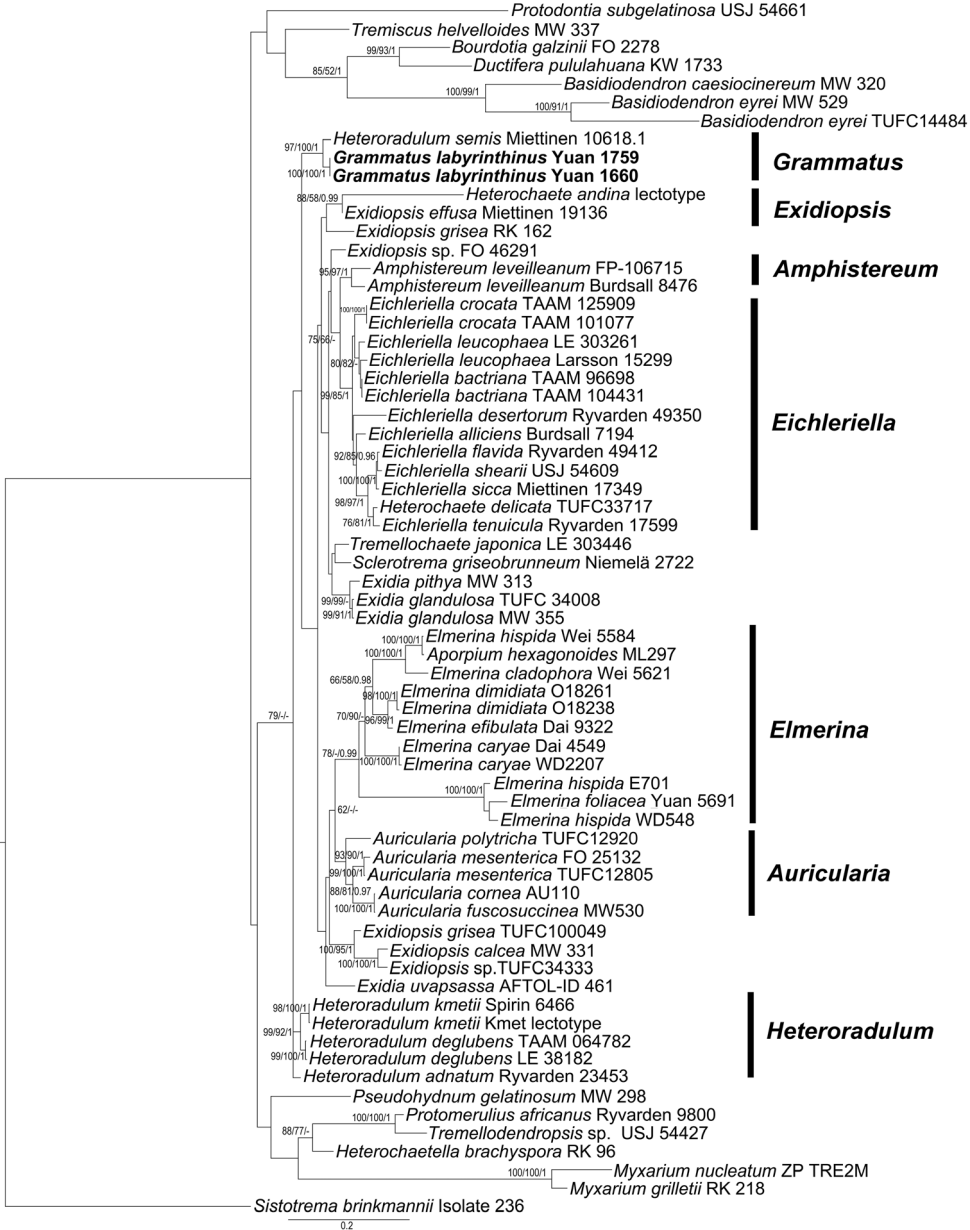
Maximum likelihood tree illustrating the phylogeny of *Grammatus labyrinthinus* and related taxa in Auriculariales, based on the combined ITS + nLSU sequence dataset. Branches are labelled with maximum likelihood bootstrap higher than 50%, parsimony bootstrap proportions higher than 50% and Bayesian posterior probabilities more than 0.95.

The combined ITS + nLSU sequence dataset includes the new species and other related species in Auriculariales. *Sistotrema brinkmannii* was used as outgroup (Malysheva and Spirin 2017). The data matrix comprised 1413 base pairs with 818 constant characters, 206 parsimony-uninformative variable characters and 389 parsimony informative positions. Maximum parsimony analysis was performed and a strict consensus tree was obtained from the 2 equally most parsimonious trees. The same dataset and alignment was analysed using RAxML v8.2.4 and MrBayes 3.2.6 with the best-fit model (GTR + I + G) selected by MrModeltest 2.3 and a similar topology was generated and the maximum likelihood tree is shown in Fig. 1. Bayesian analysis ran 4 million generations and resulted in average standard deviation of split frequencies = 0.008219. In the phylogenetic tree, two sampled specimens of *Grammatus labyrinthinus* group together with full support and form a monophyletic lineage with *Heteroradulum semis* with strong support (97 % in ML, 100 % in MP and 1.00 BPP). The new taxon belongs to Auriculariales in which it has an isolated position.

### Taxonomy

#### Grammatus

Taxon classificationFungiAuricularialesAuriculariaceae

H.S. Yuan & C. Decock gen. nov.

MB825392

##### Notes.

Basidiocarps annual, resupinate; hymenophoral surface hydnoid, irregularly poroid to labyrinthine, hymenium restricted to the area surround the spines or the bottom of the tubes; Hyphal system dimitic; skeletocystidia heavily encrusted in trama; dendrohyphidia thin- to slightly thick-walled; basidia longitudinally septate; basidiospores thin-walled, smooth, oblong-ellipsoid to cylindrical.

##### Type species.

*Grammatus labyrinthinus* H.S. Yuan & C. Decock.

##### Etymology.

*grammatus*: referring to the hymenophore striped with raised lines.

Basidiocarps annual, resupinate, coriaceous; hymenophoral surface cream to pale buff, covered by evenly distributed blunt-pointed spines or irregularly irpicoid to subporoid, then developing into labyrinthiform to sinuous pores; hymenium restricted to the area surrounding the spines or the bottom of the tubes. Subiculum very thin. Spine or tubes corky, concolorous with hymenophoral surface, shallow. Hyphal system dimitic; generative hyphae bearing clamp connections; skeletal hyphae IKI–, CB+; tissue unchanged in KOH. Skeletocystidia clavate, upper part heavily encrusted. Dendrohyphidia present. Basidia subglobose, longitudinally septate. Basidiospores oblong-ellipsoid to cylindrical, hyaline, thin-walled, smooth, IKI–, CB–.

#### Grammatus labyrinthinus

Taxon classificationFungiAuricularialesAuriculariaceae

H.S. Yuan & C. Decock sp. nov.

MB825393

Figures 3 , 4

##### Diagnoses.

Basidiocarps annual, resupinate; hymenium restricted to the base of the tubes. Hymenophoral surface irregularly irpicoid to subporoid, then labyrinthine to sinuous. Subiculum very thin. Tubes shallow. Hyphal system dimitic; generative hyphae bearing clamp connections; skeletal hyphae IKI–, CB+. Skeletocystidia clavate, the upper part heavily encrusted. Dendrohyphidia present, thin- to slightly thick-walled. Basidia subglobose, longitudinally septate. Basidiospores oblong-ellipsoid to cylindrical, hyaline, thin-walled, smooth, IKI–, CB–.

##### Type.

**China.** Yunnan Province, Xishuangbanna, Jinghong County, Nabanhe Nat. Res., fallen angiosperm branch, 17.VIII.2005 *Yuan 1759* (holotype: IFP 019121).

##### Etymology.

*labyrinthinus* (Lat.): refers to labyrinthine hymenophore.

Basidiocarps annual, resupinate, coriaceous, without special odour or taste when fresh, corky when dry, up to 15 cm long, 3 cm wide and 0.2 mm thick. Hymenophoral surface cream to pale buff when fresh, cinnamon-buff to yellowish-brown upon drying, firstly irregularly irpicoid to subporoid, the separate plates grow laterally and then develop into labyrinthine to sinuous pores, mostly 4–5 per mm, dissepiments thin; sterile margin up to 0.2 mm wide, pale yellow. Subiculum very thin (ca. 0.1 mm thick), cream to pale buff. Tubes corky, concolorous with pore surface, shallow, up to 130 µm deep, tube walls 120–200 µm thick. Hymenium restricted to the base of the tubes.

Hyphal structure. Hyphal system dimitic; generative hyphae bearing clamp connections, skeletal hyphae IKI–, CB+; tissue unchanged in KOH.

Subiculum. Dominated by skeletal hyphae; generative hyphae hyaline, thin-walled, rarely branched, 1.5–2.8 µm diam; skeletal hyphae hyaline, thick-walled to subsolid, straight to flexuous, covered by fine crystals, occasionally branched, interwoven, 1.8–3 µm diam.

Tubes. Generative hyphae infrequent, hyaline, thin-walled, rarely branched, 1.5–2.5 μm diam; skeletal hyphae dominant, hyaline, thick-walled to subsolid, moderately branched, interwoven, 1.8–2.8 μm diam. Skeletocystidia numerous, clavate, thick-walled, originating from and tightly embedded in trama, upper part heavily encrusted, 10–30 × 4–8 µm (with encrustation). Dendrohyphidia present, especially along the dissepiments, arising from generative hyphae, thin- to slightly thick-walled, apically moderately to strongly branched. Basidia subglobose, longitudinally septate, already septate as probasidia, 18–25 × 10–13 μm, epibasidia divided into four parts up to 20 μm long, bearing four sterigmata and without clamp connection at the base, sterigmata up to 20 μm long.

Basidiospores. Oblong-ellipsoid to cylindrical, hyaline, thin-walled, smooth, IKI–, CB–, (13–)13.3–15.7(–16) × (6–)6.4–7.4(–7.7) μm, L = 14.4 μm, W = 6.94 μm, Q = 2.07–2.1 (n = 60/2).

##### Type of rot.

White rot.

Additional specimens examined – **China.** Yunnan Province, Xishuangbanna, Jinghong County, Elephant Valley Forest Park, fallen angiosperm branch, 14.VIII.2005 *Yuan 1600* (IFP 019118); Nabanhe Nat. Res., fallen angiosperm branch, 15.VIII.2005 *Yuan 1683* (IFP 019119); fallen angiosperm branch, 17.VIII.2005 *Yuan 1734* (IFP 019120).

#### Grammatus semis

Taxon classificationFungiAuricularialesAuriculariaceae

(Spirin & Malysheva) H.S. Yuan & C. Decock comb. nov.

MB825394

##### Basionym.

*Heteroradulum semis* Spirin & Malysheva, in Malysheva & Spirin, Fungal Biology 121: 712. 2017.

**Figure 2.**
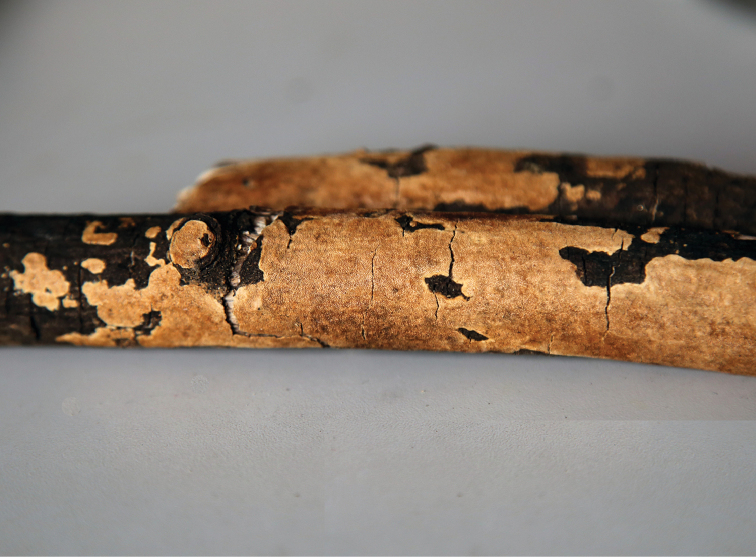
Basidiocarps of *Grammatus labyrinthinus* (*Yuan 1734*).

**Figure 3.**
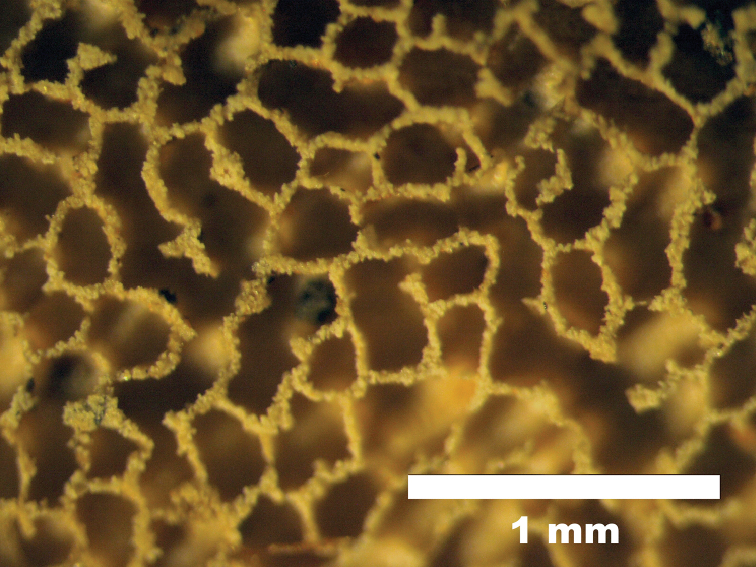
Hymenophoral surface of *Grammatus labyrinthinus* under ×8 lens (holotype).

**Figure 4.**
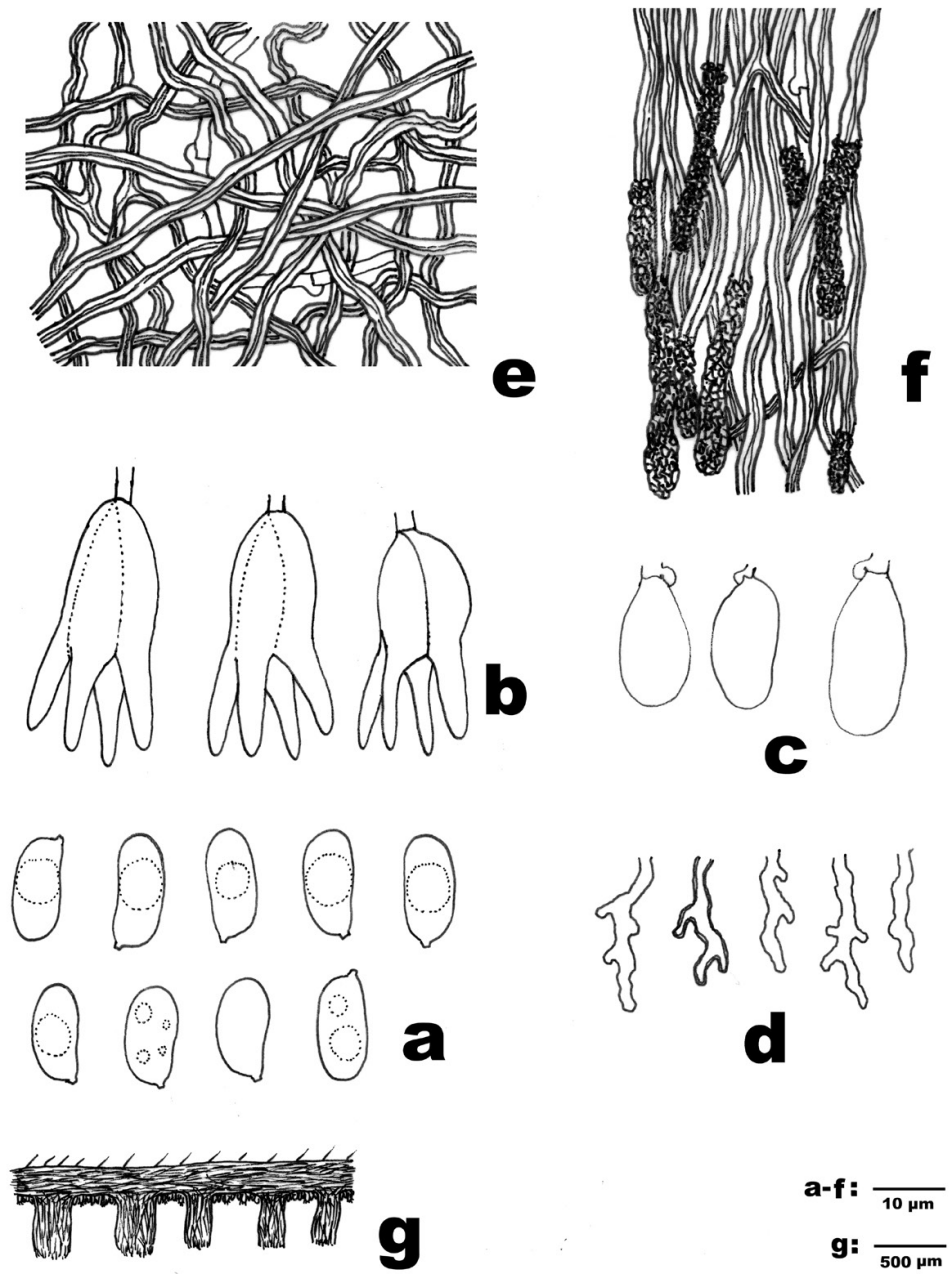
Microscopic structures of *Grammatus labyrinthinus* (drawn from the holotype). **a** Basidiospores **b** Probasidia **c** Probasidia transection **d** Epibasidia **e** Dendrohyphidia **f** Hyphae from subiculum **g** Hyphae from trama **h** Basidiocarp transection.

## Discussion

Anatomically, the longitudinally septate basidia of *Grammatus labyrinthinus* point toward affinities with Auriculariales, which is confirmed by molecular data. The new taxa are phylogenetically closely related to *Heteroradulum*. *Heteroradulum kmetii*, type of the genus, has perennial, effused-reflexed and pinkish or reddish basidiocarps with hymenial surface first smooth then with irregularly arranged, sharpened outgrowths (Malysheva and Spirin 2017), in which feature, it differs from *G. labyrinthinus*. The similarity between the ITS sequences of *G. labyrinthinus* and *H. kmetii* are of 89.84%. The unique morphological characteristics and molecular sequence analyses both support the establishment of the new genus.

*Heteroradulum semis* was originally found and described from high elevation temperate north-eastern China. It is characterised by resupinate, leathery basidiocarps covered by blunt-pointed spines, a dimitic hyphal structure with clamped generative hyphae, encrusted tramal skeletocystidia, simple or sparsely branched dendrohyphidia, longitudinally septate basidia and broadly cylindrical to narrowly obovate basidiospores (Malysheva and Spirin 2017). The sterile and blunt-pointed outgrowths on the hymenial surface of *H. semis* identify the irregularly irpicoid and separate plates of *G. labyrinthinus* when young, but the irregularly irpicoid and separate plates of *G. labyrinthinus* would form the labyrinthine or subporoid structure when old and can be distinguished from the former species. Phylogenetic analyses confirm that *H. semis* clustered with *G. labyrinthinus* with strong support. So it is transferred to *Grammatus* and a new combination, *G. semis* is proposed.

*Aporpium*, *Elmerina* and *Protomerulius* Möller all have a poroid hymenophore (Ryvarden and Johansen 1980, Núñez and Ryvarden 2001, Sotome et al. 2014). However, they are all distant from *Grammatus labyrinthinus* in phylogenetic inferences (Fig. 1).

There are 216 genera and more than 1800 species of wood-inhabiting fungi in Polyporales (Kirk et al 2008) with high diversity of hymenophore structure from smooth, hydnoid, lamellate and poroid. Amongst the poroid taxa, *Grammothele* Berk. & M.A. Curtis, *Hymenogramme* Mont. & Berk., *Porogramme* (Pat.) Pat. and *Theleporus* Fr. are characterised by a hymenium restricted to the bottom of the pores, which differentiate them from the other typical polypores. These genera are characterised by non-septate basidia and are members of Polyporales. In comparison, Auriculariales are relatively poor in genera and species, but still, their hymenophore structures are diverse. *Grammatus labyrinthinus* is the representative of poroid species with a hymenium restricted to the bottom in Auriculariales. It is another instance of morphological convergent evolution across the order.

## Supplementary Material

XML Treatment for Grammatus

XML Treatment for Grammatus labyrinthinus

XML Treatment for Grammatus semis
